# Miniature Pulpotomy of Symptomatic Mature Permanent Teeth: A Report of Two Cases

**DOI:** 10.7508/iej.2016.01.015

**Published:** 2015-12-24

**Authors:** Saeed Asgary, Mahdieh Nourzadeh, Mohammad Jafar Eghbal

**Affiliations:** a * Iranian Center for Endodontic Research, Research Institute of Dental Sciences, Dental School, Shahid Beheshti University of Medical Sciences, Tehran, Iran;*; b *Dental Research Center, Research Institute of Dental Sciences, Dental School, Shahid Beheshti University of Medical Sciences, Tehran, Iran*

**Keywords:** Calcium-Enriched Mixture, CEM Cement, Endodontic, Miniature Pulpotomy, Irreversible Pulpitis

## Abstract

Human dental pulp inflammation can progress to periapical lesion formation and conventional root canal treatment (RCT) has been the traditional method for disease management. This observational study presents two cases of vital pulp therapy in mature molars diagnosed with irreversible pulpitis and associated with apical periodontitis. In these two clinical cases, the involved teeth had deep carious lesions with a history of spontaneous/lingering pain and radiographic examinations revealed the presence of apical radiolucencies. A conservative miniature pulpotomy (MP) using calcium-enriched mixture (CEM) was performed and the teeth were permanently restored with amalgam. Clinical evaluations indicated resolution of pain 24 hours after treatment; the teeth showed normal vitality, remained asymptomatic and maintained normal function after recall examinations. Furthermore, the 18-month radiographic evaluation showed healing of the apical lesions. Vital pulp therapy using the MP technique with CEM appeared successful in avoiding RCT intervention. These two reports of case outcome suggest that simple MP using a CEM bioregenerative technique may provide a favorable outcome for permanent teeth diagnosed with irreversible pulpitis and associated with apical periodontitis.

## Introduction

Dental caries can promote mild-to-severe inflammation of the dental pulp. Irreversible pulpitis is an inflammatory condition of the dental pulp characterized by long lasting or lingering pain after removal of a specific stimulus. If this inflammatory process progresses, immune responses in the periapical region, can advance and eventually lead to the development of apical periodontitis. It has been demonstrated that periradicular lesions may be detected before the actual necrosis of dental pulp [1]. Scientific evidence supports the probability that a symptomatic carious tooth with a periapical lesion may exhibit a vital dental pulp; therefore, apical periodontitis does not always indicate a necrotic pulp [1-4]. In the new millennium, the ultimate goal of endodontology should be to reinstate the diseased or necrotic human dental pulp to a normal state of health and function [5] without complex intervention *i.e.* root canal therapy (RCT) [6]. 

Vital pulp therapy (VPT) is a simple, biologic, regenerative, conservative and economic method that shows a favorable success rate; treatment includes direct/indirect pulp capping and miniature/partial/complete pulpotomy using various pulp capping agents [7, 8]. Miniature pulpotomy (MP) is a procedure that requires minimal removal of the infected dentin/injured pulp tissue not exceeding 1 mm at the exposure site. This type of VPT creates a clean surgical wound and enhances interaction of the pulp capping agent at the undifferentiated mesenchymal or dental pulp stem cell interface [9]. 

Mineral trioxide aggregate (MTA), which has the ability to induce hard tissue formation, is universally used in VPT [10] but it has some disadvantages that include long setting time, difficult handling characteristics, potential for tooth discoloration and higher cost [11]. It has been shown that calcium enriched mixture (CEM) which exhibits several advantageous physico-chemical properties, can provide favorable outcomes in VPT for permanent teeth diagnosed with irreversible pulpitis [12].

**Figure 1:**
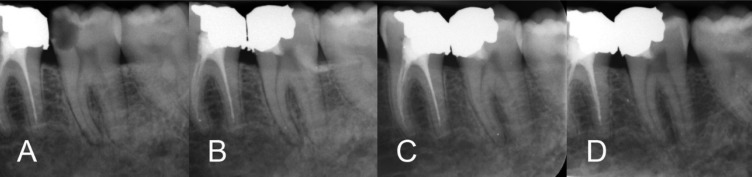
*A)* preoperative intraoral x-ray*; B) *immediate postoperative x-ray;* C) *12-month follow-up;* D) *18-month follow-up

**Figure 2:**
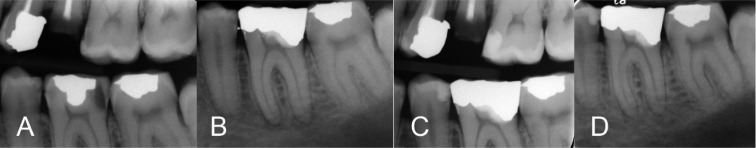
*A)* preoperative x-ray; *B)* immediate postoperative x-ray; *C *and *D)* 12-month follow-up bitewing and periapical radiographies

This clinical report describes successful MP treatment of two mandibular molars diagnosed with irreversible pulpitis and associated with periapical involvement using CEM cement. 

## Case Report

***Case 1***

A 38-year-old female presented to our clinic with the chief complaint of spontaneous and severe lingering pain provoked by chewing and exposure to cold fluids. The patient’s medical history was noncontributory and clinically, the left mandibular second molar exhibited a deep carious lesion. The involved tooth responded with prolonged exaggerated pain (>10 seconds) to a cold test and demonstrated sensitivity to percussion. Radiographic examination revealed a deep carious lesion and complete root-end apical closure, widening of periodontal ligament and presence of a periapical lesion (Figure 1A). According to conventional clinical and radiographic findings, the diagnosis would be irreversible pulpitis and symptomatic apical periodontitis.

A mouth rinse with 0.2% chlorhexidine gluconate was first initiated and the patient then received inferior alveolar nerve block anesthesia with 2% lidocaine and 1:80000 adrenalin (Darupakhsh, Tehran, Iran). The tooth was isolated with a dental dam and then caries were completely excavated under magnification. The exposed surface of the dental pulp was lightly brushed with a sterile round diamond bur (Komet, Lemgo Switzerland) using a high-speed handpiece and copious water irrigation; the removal of the pulp did not exceed 1 mm. Hemostasis was achieved by irrigation with sterile saline solution and gentle application of moistened sterile cotton pellets for three min. CEM cement powder and liquid (BioniqueDent, Tehran, Iran) were mixed according to the manufacturer’s instructions. A small bulk of the cement was gently condensed into the pulpal cavity using dry sterile cotton pellets. After 5 min [1], the prepared cavity was carefully restored with amalgam [2] and an immediate post-operative radiograph taken (Figure 1B). After the first follow-up session at 24 h, the tooth was considered asymptomatic and longer-term follow-ups evaluations demonstrated normal vitality to pulp testing, the tooth remained functional and asymptomatic. Intraoral periapical radiographs showed formation of a calcific bridge below the CEM biocement and normal periodontal ligament space width with remineralization of the osseous defect at the 12- and 18-month follow-ups (Figures 1C to D).

***Case 2***

A healthy 40-year-old man was referred to our clinic with a chief complaint of spontaneous and temperature related pain including pain on chewing in the left mandible. Clinically, the mandibular left first molar exhibited an advanced distal carious lesion (Figure 2A) and was sensitive to cold/percussion testing. Similar to first case, after a mouth rinse with 0.2% chlorhexidine gluconate, the tooth was anesthetized and isolated. After complete caries removal, the exposed pulp was removed gently with a sterile round high-speed diamond bur; the total removal of the pulp did again not exceed 1 mm. Hemorrhage was controlled and CEM cement was placed on the exposure site and surrounding dentin with 2 mm thickness; the tooth was restored with amalgam and post-operative radiography was taken (Figure 2B).

The patient was recalled after one week and the tooth was clinically asymptomatic. The tooth showed normal vitality to cold testing, with no evidence of clinical or radiographic pathology at the 18-month recall. An important radiographic finding was formation of a hard tissue bridge beneath CEM cement and complete resolution of the apical lesions (Figures 2C to D).

## Discussion

As our knowledge in pulpal biology and endodontic biomaterials advances, the initial management of vital teeth with associated apical lesions employing simple VPT procedures may now be considered a potential treatment strategy. VPT is a valuable treatment, particularly for teeth with open apices and vital pulps which are mechanically or cariously exposed [13-15]. Usually, permanent mature teeth with irreversible pulpitis with/without the presence of apical periodontitis are treated by RCT. However, investigations have shown a lower survival rate of endodontically treated teeth when compared to their vital counterparts [16, 17]. Currently, randomized clinical trials have reported successful outcomes for VPT in symptomatic permanent teeth with cariously exposed pulps with or without periapical lesions [4, 7, 12, 18-20]. The goals of VPT include preservation of dental pulp vitality and to encourage repair of the remaining pulp tissue to re-establish structural and functional health of the pulp-dentin complex [21, 22]. The response of the dental pulp to VPT can include the formation of fibrodentin, calcific tissues, reparative dentin and remineralization of the surrounding dentin. This process is generated through the recruitment of odontoblast-like cells or differentiation and proliferation of dental pulp stem cells [23]. The advantages of VPT over RCT include decreased clinical time and cost, less tooth destruction, preservation of pulp vitality and proprioceptive mechanisms, higher survival rates, and less postoperative pain and complications [7]. Moreover, in cases where VPT may be unsuccessful, the tooth can be managed by complete pulpotomy, regenerative endodontics or conventional RCT.

In this report, two cases with irreversible pulpitis and symptomatic apical periodontitis were treated by MP using CEM bioceramic. Follow-up examinations demonstrated favorable outcomes that maintained pulpal vitality and promoted periapical healing using CEM as a VPT bioceramic. The biological response to VPT is highly influenced by the healing potential of the inflamed pulp as well as the biocompatibility and sealing ability of pulp capping agent [12]. The successful outcome of VPT in teeth with irreversible pulpitis and periapical lesions has been demonstrated in other investigations and have underscored the importance of the regenerative and repair ability of dental pulp stem cells [3, 24, 25]. Another study conducted to evaluate the outcome of full pulpotomies using CEM in symptomatic permanent molars. Radiographic findings showed that, in cases with periapical involvement, eliminating the etiological factors resulted in periapical healing in 91% of cases after 1-year follow-ups [12]. Investigators reported the formation of normal periradicular bone, following the VPT/CEM of a mature mandibular molar with irreversible pulpitis and associated condensing apical periodontitis [12].

MP is a conservative VPT strategy, which results in removal of injured odontoblast cells, operative debris, potentially infected tissue and exposes deeper layers of the pulp to the proximity of the capping agent where mesenchymal or dental pulp stem cells are present. The advantages of MP include better irrigation and cleaning of the surgical pulp wound from contaminated dentinal chips and accessibility to areas with less pulpal inflammation where hemostasis can be more easily attained. The procedure also creates adequate space for the suitable pulp capping agent or biomaterial, thus producing a superior three dimensional antibacterial seal [9]. 

Current research data indicates that the hard tissue barriers generated under CEM cement are thicker than those produced by MTA and that the layer of odontoblast-like cells formed underneath the capping material appears to be more consistent in CEM specimens [12]. It has been shown that the pulpal reaction to CEM cement is similar to MTA and that these two bioceramic materials have the same biological characteristics when compared as pulp capping agents [12]. Another recent clinical report has also shown that CEM has the ability to induce the formation of a contiguous calcified bridge in the presence of injured dental pulps after miniature pulpotomy [26].

The mechanism by which CEM encourages pulp healing still remains unclear. However, this feature may be attributed to several properties including sealing ability, antimicrobial effect, low cytotoxicity, similarity to dentine, hydroxyapatite formation and the ability to induce hard barrier formation [12, 27-30]. 

## Conclusion

Miniature pulpotomy provides an easy, conservative, and biologically-based treatment that maintains pulpal vitality and shows the ability to resolve emerging apical pathosis under certain conditions. Although more clinical trials with larger sample sizes and longer-term follow-ups may be required, using CEM cement or other bioceramic materials for MP may be considered an alternative option to conventional RCT in symptomatic vital permanent teeth diagnosed with irreversible pulpitis.
